# MicroRNA Expression Profiles in Gastric Carcinogenesis

**DOI:** 10.1038/s41598-018-32782-8

**Published:** 2018-09-26

**Authors:** Jinha Hwang, Byung-Hoon Min, Jiryeon Jang, So Young Kang, Hyunsik Bae, Se Song Jang, Jong-Il Kim, Kyoung-Mee Kim

**Affiliations:** 1 Department of Biomedical Science, Seoul National University Graduate School, Seoul, Korea; 2 Department of Medicine, Samsung Medical Center, Sungkyunkwan University School of Medicine, Seoul, Korea; 3 Department of Health Sciences and Technology, SAIHST, Sungkyunkwan University, Seoul, Korea; 4 Department of Pathology and Translational Genomics, Samsung Medical Center, Sungkyunkwan University School of Medicine, Seoul, Korea; 5 Cancer Research Institute, Seoul National University College of Medicine, Seoul, Korea; 6 Genomic Medicine Institute, Medical Research Center, Seoul National University, Seoul, Korea

## Abstract

Intestinal-type gastric carcinoma exhibits a multistep carcinogenic sequence from adenoma to carcinoma with a gradual increase in genomic alterations. But the roles of microRNAs (miRNA) in this multistage cascade are not fully explored. To identify differentially expressed miRNA (DEM) during early gastric carcinogenesis, we performed miRNA microarray profiling with 24 gastric cancers and precursor lesions (7 early gastric cancer [EGC], 3 adenomas with high-grade dysplasia, 4 adenomas with low-grade dysplasia, and 10 adjacent normal tissues). Alterations in the expression of 132 miRNA were detected; these were categorized into three groups based on their expression patterns. Of these, 42 miRNAs were aberrantly expressed in EGC. Five miRNA (miR-26a, miR-375, miR-574-3p, miR-145, and miR-15b) showed decreased expression since adenoma. Expression of two miRNA, miR-200C and miR-29a, was down-regulated in EGCs compared to normal mucosa or adenomas. Six miRNA (miR-601, miR-107, miR-18a, miR-370, miR-300, and miR-96) showed increased expression in gastric cancer compared to normal or adenoma samples. Five representative miRNAs were further validated with RT-qPCR in independent 77 samples. Taken together, these results suggest that the dysregulated miRNA show alterations at the early stages of gastric tumorigenesis and may be used as a candidate biomarker.

## Introduction

A subset of gastric cancer (intestinal-type gastric cancer by Lauren’s histologic classification) exhibits a gradual development through a multistep carcinogenic sequence from non-neoplastic atrophic and metaplastic gastric mucosa to adenoma with low-grade dysplasia (LGD), followed by adenoma with high-grade dysplasia (HGD) and eventually develops into invasive carcinoma^1,2^. A recent report by Min *et al*. showed a gradual increase in genomic alterations, including somatic nucleotide variation, gene fusion, and copy number variation, from LGD to carcinoma^3^.

Aside from the accumulation of genetic alterations, epigenetic changes such as DNA methylation and aberrant gene expression by non-coding RNAs are other key players involved in carcinogenesis^4^. MicroRNAs (miRNAs) are abundant non-coding RNA molecules of 18–25 nucleotides that inhibit translation or promote degradation of messenger RNAs (mRNAs) with complementary sequences. miRNAs are estimated to regulate the expression of 30–60% of human genes and are known to modulate cell development, differentiation, proliferation, and apoptosis. Hence, alterations in their expression are associated with human diseases such as cancer^5,6^. Depending on its mRNA target, the miRNA may function as a tumor suppressor or promoter of tumorigenesis^7^.

In colorectal and esophageal adenocarcinomas, miRNA profiles of cancer and their precursor lesions such as Barrett’s esophagus or colorectal adenoma are well described^8–15^. These profiles have the potential to be used as diagnostic biomarkers for early cancer detection and therapeutic targets for cancer prevention. Only a single study has reported the gradual increase in miR-106a, a member of miR-17 family, during the multistep gastric carcinogenesis using real-time quantitative polymerase chain reaction (RT-qPCR)^16^. However, there are no reports on miRNA profiles in gastric precursor lesions or during multistep carcinogenesis.

To identify miRNA expression signatures through a multistep carcinogenic sequence, we performed NanoString miRNA expression assays in normal gastric mucosa, LGDs, HGDs, and intestinal-type early gastric cancers (EGCs) and subsequently validated five miRNAs using independent sample sets with RT-qPCR. In addition, we used RNA sequencing data from our previous study to investigate the correlation between the expression of miRNAs and their target mRNAs.

## Results

### Altered miRNA expression in normal gastric mucosa, adenoma, and EGC

We performed miRNA expression arrays by using NanoString to identify DEM patterns in normal gastric mucosa, adenoma with LGD and HGD, and EGC (Table 1). We identified 132 DEMs among three groups (p-value < 0.01, average expression >5, and fold change > = 2.5 or < = 0.4). DEMs were categorized into three groups based on the changing patterns of miRNA expression. The DEM-1 group exhibited a decrease in the miRNA expression from adenoma lesion (DEM-1a, n = 16) or EGC (DEM-1b, n = 7) compared to normal gastric mucosa. DEM-2 group comprised cases with highest miRNA expression levels in adenomas (n = 3). DEM-3 group showed an upregulated miRNA expression in EGC as compared with normal mucosa or adenomas (n = 98) (Supplementary Table 1).

**Table 1:** Demographics of the discovery cohorts.

Pathology	Sample No.	Gender	Age	Size (cm)	Invasion Depth	Lymphatic invasion	*H. pylori* infection	EBV infection
EGC	CST04	M	75	1.2	LP	No	Yes	No
EGC	CST95	M	63	1.8	LP	No	Yes	No
EGC	CST22	F	69	4.2	LP	No	Yes	No
EGC	CST36	M	75	2.2	MM	No	Yes	No
EGC	CST58	F	65	3.0	MM	No	Yes	No
EGC	CST78	M	66	3.2	MM	No	Yes	No
EGC	CST29	M	72	4.5	MM	No	Yes	No
HGD	HST92	F	74	0.4			Yes	No
HGD	HST87	M	64	1.4			Yes	No
HGD	HST85	M	63	2.3			No	No
LGD	LST09	F	63	1.1			Yes	No
LGD	LST34	F	75	2.1			Yes	No
LGD	LST20	M	74	2.6			Yes	No
LGD	LST72	M	64	3.0			Yes	No

**Figure 1:**
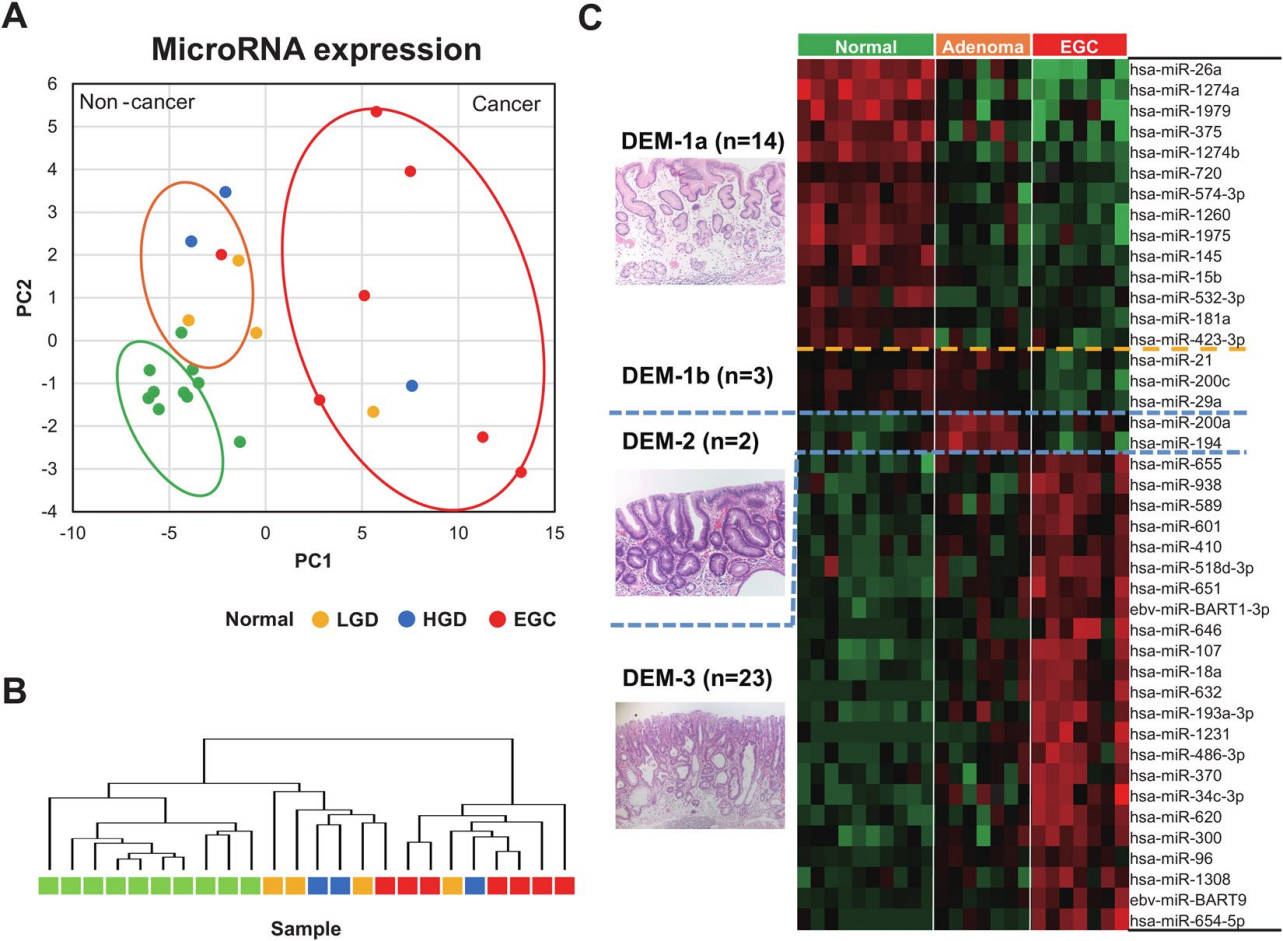
The expression profiles of miRNA for LGD, HGD, and EGC. (**A**) Principal component analysis of the whole set of miRNAs. (**B**) Results of un-supervised clustering analysis. (**C**) Differentially expressed miRNAs (DEM) among three groups. DEM-1 with highest expression levels in normal control mucosa, DEM-2 with highest miRNA expression levels in adenoma, and DEM-3 with upregulated miRNA in EGC with their representative histopathologic findings.

Principal component analysis (PCA) showed that the cancer and non-cancer samples were separated along the PCA1 axis, whereas the normal gastric mucosa and adenoma samples were differentially located along the PC2 axis (Fig. 1A). Unsupervised PCA results showed that normal and adenoma samples showed similar expression patterns, but EGC samples tended to cluster to a separate group (Fig. 1B).

We found that 42 miRNAs were aberrantly expressed (false discovery rate [FDR] <0.01) in early gastric tumorigenesis (Fig. 1C). Among the 42 DEMs, we selected 13 miRNAs that showed patterns identical or similar to those reported in previous miRNA studies (Table 2). In comparison to normal gastric mucosa samples, adenoma samples showed a decrease in the expression of five miRNAs (miR-26a, miR-375, miR-574-3p, miR-145, and miR-15b), which are known to be down-regulated in gastric cancer^17–21^. The expression of two miRNAs (miR-200C and miR-29a), which are known to be down-regulated in gastric cancer^22,23^, decreased in EGCs as compared with normal mucosa or adenomas. In addition, there was an increase in the expression of six miRNAs (miR-601, miR-107, miR-18a, miR-370, miR-300, and miR-96), which are known to be upregulated in gastric cancer^24–35^, in EGCs as compared with normal or adenoma samples (Fig. 1C and Table 2).

**Table 2:** Differentially expressed miRNAs showing identical patterns as in the previous miRNA studies.

DEM group	miRNA	Fold change adenoma/normal	Fold change egc/normal	Fold change egc/adenoma	FDR	Up- or down- regulation	Reference
DEM-1a	hsa-miR-26a	0.33	0.07	0.22	5.25E-05	Down	^17^
	hsa-miR-375	0.45	0.15	0.33	9.73E-03	Down	^18, 46– 48^
	hsa-miR-574-3p	0.32	0.26	0.80	2.58E-04	Down	^19^
	hsa-miR-145	0.24	0.20	0.85	1.98E-03	Down	^20^
	hsa-miR-15b	0.48	0.47	0.98	1.98E-03	Down	^21^
DEM-1b	hsa-miR-200c	0.73	0.26	0.36	4.43E-03	Down	^22^
	hsa-miR-29a	0.91	0.39	0.43	4.60E-03	Down	^23^
DEM-3	hsa-miR-601	2.53	4.69	1.85	1.98E-03	Up	^24^
	hsa-miR-107	2.98	6.31	2.12	5.14E-03	Up	^25– 28, 41^
	hsa-miR-18a	*2.03*	*3.46*	1.70	5.89E-03	Up	^29– 31^
	hsa-miR-370	*2.02*	*5.65*	2.80	4.38E-03	Up	^32, 33^
	hsa-miR-300	*2.13*	*4.58*	2.15	1.98E-03	Up	^34^
	hsa-miR-96	*1.92*	2.62	1.36	3.23E-03	Up	^35^

Additionally, we validated two novel miRNAs which have been established as tumor suppressor or suppressor of epithelial-to-mesenchymal transition (miR-655) in hepatocellular^36^ and esophageal squamous cell carcinoma^37,38^, and reported as oncogenic (miR-938) miRNA in colorectal cancers^39^, but their roles have not been studied in gastric cancer. Unexpectedly, we found that expressions of miR-655 decreased from normal to adenoma and carcinoma, suggesting tumor suppressive role in gastric cancer. Meanwhile, miR-938 was up-regulated in EGCs compared to normal gastric mucosa and adenomas, suggesting oncogenic function in gastric cancer (Supplementary Fig. 1).

### Validation of results and correlation between miRNA and target genes

**Figure 2:**
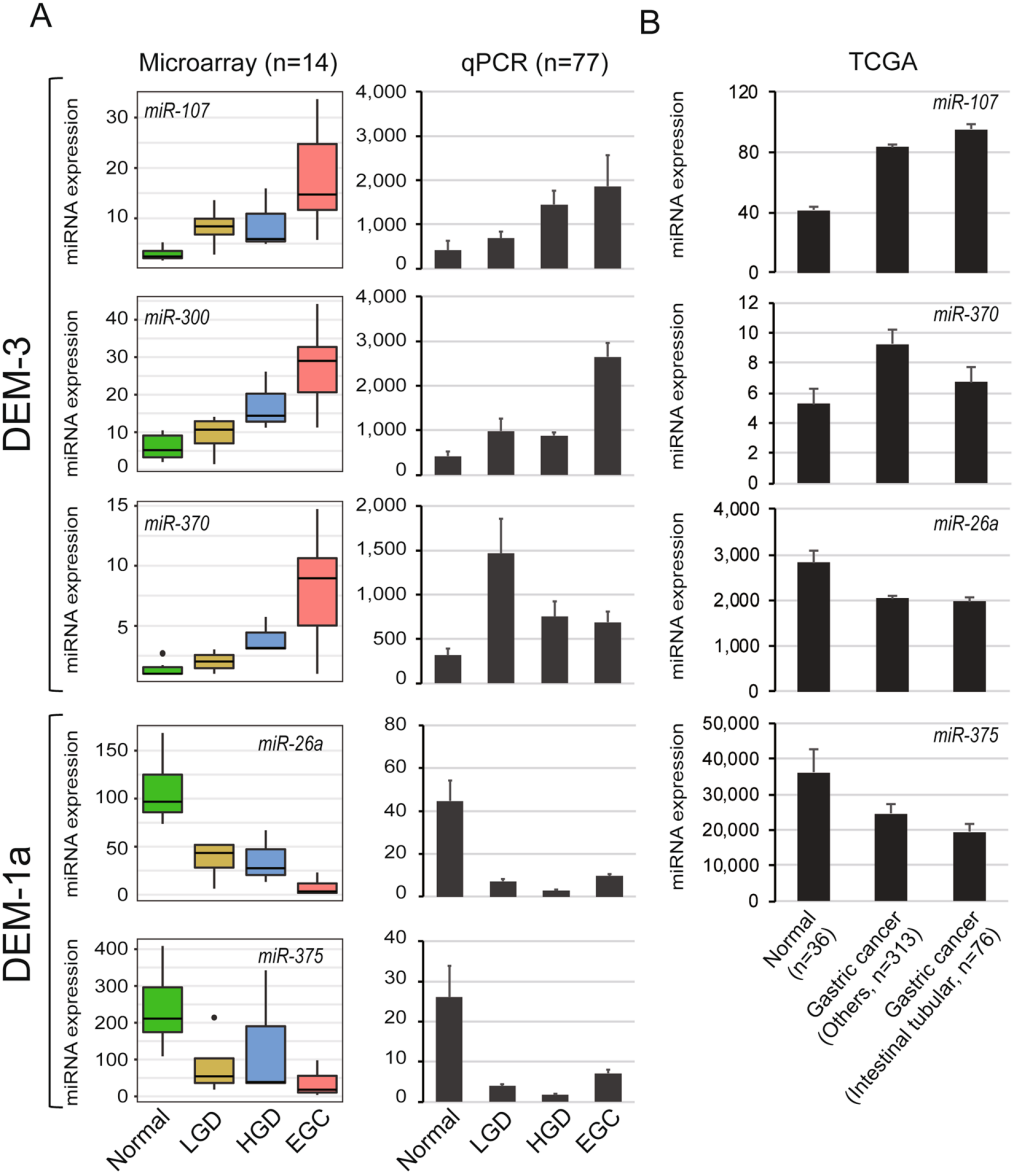
Stepwise changes in the expression of miRNAs during early gastric carcinogenesis. (**A**) The expression levels of five miRNAs were measured using miRNA array and real-time RT-PCR. (**B**) The expression patterns of four miRNAs were also identified in TCGA dataset.

For further experimental validation, we measured the expression of five miRNAs (miR-107, miR-300, and miR-370 from DEM-3; miR-26a and miR-375 from DEM-1a) in 77 independent samples using RT-qPCR, owing to the obvious difference (fold change >4) in their expression between normal and EGC group in the miRNA microarray analysis. We observed a progressive increase in the expression of miR-107 and miR-300 from normal to adenoma and EGC in microarray analysis; these results were subsequently validated in an independent sample set with RT-qPCR. In comparison to the normal gastric mucosa, adenomas and EGCs showed a decrease in the expression of miR-26a and miR-375 in both microarray and qPCR assays (Fig. 2A). However, we failed to confirm the gradual increase in the expression of miR-370 in the validation set. From TCGA datasets, we found that 4 miRNAs (miR-107, miR-370, miR-26a and miR-375) were dysregulated in gastric cancer compared to normal gastric mucosa and also showed differential gene expression levels between tubular adenocarcinoma (well- and moderately differentiated, intestinal-type by Lauren) group (n = 76) and other types of gastric cancer group (n = 313) (Fig. 2B).

**Figure 3:**
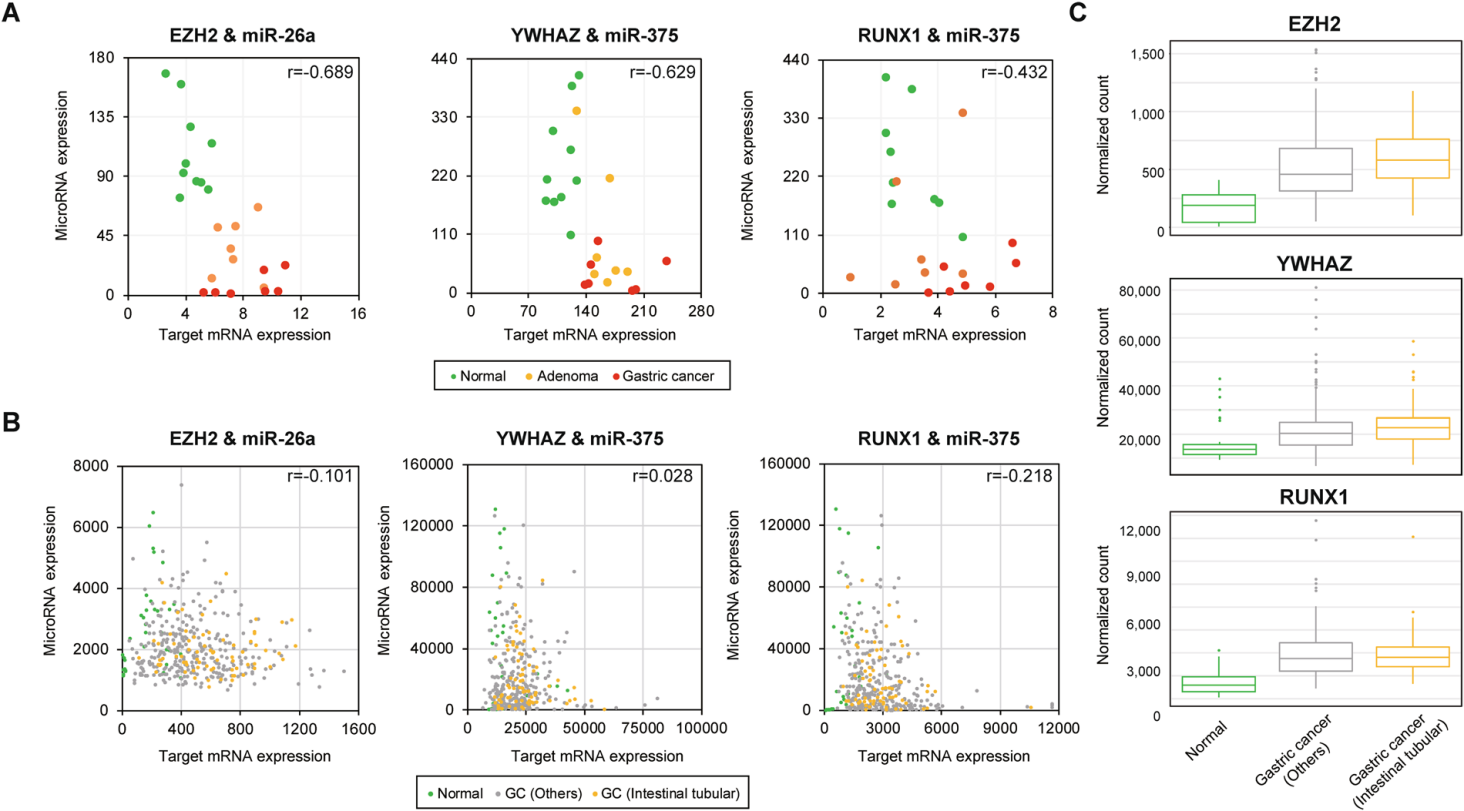
Spearman’s correlation between miRNA and well-known target genes in samples from normal, LGD, HGD, and EGC. (**A**) Negative correlation between miR-26a and *EZH2* (***P = 2.87E-4, r = -0.689). Negative correlation between miR-375 and *YWHAZ* (*P = 1.28E−3, r = −629) and *RUNX1* (*P = 3.61E−2, r = −0.432) in microarray data. (**B**) Negative correlation between miR-26a and *EZH2* (*P = 2.18E-2, r = -0.101). Negative correlation between miR-375 and *YWHAZ* (P = 0.288, r = 0.028) and *RUNX1* (***P = 5.00E-6, r = -0.218) observed in TCGA data. (**C**) Expression of miRNA and their target gene mRNA expression patterns in TCGA dataset.

To determine the impact of these miRNAs on target genes, we calculated the correlation coefficient between the expression of miRNAs and their target genes using our previous RNA sequencing data. A negative correlation was observed between miR-26a and EZH2 (r = −0.689), miR-375 and YWHAZ (r = −0.629), and miR-375 and RUNX1 (r = −0.432) (Fig. 3A). We also found weak negative correlation between miR-26a and EZH2 (r = −0.101), miR-375 and RUNX1 (r = −0.218) from TCGA dataset (Fig. 3B). Three target genes (EZH2, YWHAZ and RUNX1) were upregulated in gastric cancer compared to normal gastric mucosa. Moreover, their normalized mRNA levels also showed slight difference between well- and moderately differentiated tubular, intestinal-type gastric cancer group compared to other types of cancers (Fig. 3C). We also performed correlation analyses with miR107, miR300, and miR370 with their target genes and only miR375 and miR26a showed high correlations with their target genes (Supplementary Fig. 2).

## Discussion

The pathogenesis of gastric cancer involves multistep genetic and epigenetic alterations, which predispose cells to neoplastic transformation^1–3^. Given the importance of miRNAs in the regulation of cell growth and viability, miRNA dysregulation is believed to be closely correlated with the development and progression of gastric cancer^4^. In the stomach, previous research on miRNA dysregulation was focused on gastric cancer itself^6,7,18,20,22,23,26,27,29,33,40^ and only one study has been conducted to explore the role of miRNA during the histologic progression from gastric adenoma to carcinoma without non-tumorous controls, warranting more research^16^. To our best knowledge, this is the first study to evaluate and compare the expression profile of miRNAs in non-tumor tissue, LGD, HGD, and EGC, using a high-throughput screening array. The aim of our study was to screen alterations in miRNA expressions during the stepwise gastric carcinogenesis and we demonstrated the role of miRNAs during the stepwise gastric carcinogenesis and confirmed their expressions with RT-qPCR.

Although previous reports have revealed several molecular alterations in gastric cancer such as DNA mutations, copy number variations, mRNA expression changes, and miRNA alterations, most of these studies were conducted by comparing gastric cancer tissues or cell lines with normal gastric samples, without adenoma samples. Zhu *et al*.^16^ investigated the miR-106a expression in gastric dysplasia and gastric samples using *in situ* hybridization. The frequency and extent of miR-106a expression was found to show a gradual increase along the histologic progression from mild, moderate, and severe dysplasia to EGC. However, they failed to evaluate the expression of other miRNAs. In the present study, we identified alterations in miRNA expressions using normal, LGD, HGD, and EGC samples. Overall, the expression of miRNAs showed distinct patterns as compared with the expression pattern of mRNAs. In comparison to the EGC samples, samples from the non-cancerous group (normal and adenoma samples), showed similar miRNA expression pattern. Within the non-cancerous group, the normal and adenoma samples were separated based on their expression patterns.

The expression of miR-107 has been reported to increase in the serum and tissues of gastric cancer patients and gastric cancer cell lines. miR-107 acts as an oncogene and regulates gastric cancer development and progression by targeting *NF1*, *DICER1*, *FOXO1*, and *CDK8*^25,27,28,41^. We found a gradual increase in the expression of miR-107 with the histologic progression from LGD to EGC. Microarray and RT-qPCR results revealed that the expression of miR-107 was significantly upregulated in HGD and EGC as compared with the normal mucosa (**p = 0.006 and *p = 0.031, respectively).

Overexpression of miR-300 promotes cell cycle progression, cell proliferation, and invasion in several cancers, including gastric cancer^34^, liver cancer^40^, and osteosarcoma^42^. miR-300 displays the potential to be used in the treatment, diagnosis, and prognosis of gastric cancer, as its expression decreased in gastric cancer following chemotherapy or exposure to radiation^43^.

In gastric cancer, miR-370 showed altered expression and acted as either an oncogene or a tumor suppressor. Previous studies have shown that the up-regulation of miR-370 resulted in the progression of gastric carcinoma via suppression of transforming growth factor beta receptor II (TGFβRII) or *FOXO1*^32,33^. Although we observed a relative increase in the expression of miR-370 in LGD, it showed a weak correlation with the expression of its target gene, *FOXO3*.

The expression of miR-26a is frequently aberrant in many tumors such as gastric cancer, bladder tumor, breast cancer, oral squamous cell carcinoma, and Burkitt lymphoma^44^. miR-26a is known to be significantly down-regulated in gastric cancer and suppresses tumor growth and metastasis by targeting *FGF9* gene^17^. Furthermore, miR-26a improves the sensitivity of gastric cancer cells to cisplatin-based chemotherapies by targeting *NRAS* and *E2F2*^45^. We found a decrease in the expression of miR-26a from LGD and a strong negative correlation between the expression of miR-26a and its known target *EZH2* (r = −0.689).

Previous studies have demonstrated that miR-375 inhibits cell proliferation of gastric cancer cells by repressing *JAK2*, *ERBB2*, and *YWHAZ*^18,46–48^. We found a significant decrease in miR-375 expression in LGD, HGD, and EGC, as evident from the microarray and RT-qPCR results. Moreover, miR-375 expression was inversely correlated (r = −0.629) with the expression of *YWHAZ* (14-3-3ζ), a member of the 14-3-3 family of proteins, which has been implicated in the initiation and progression of cancers and is a potential biomarker for gastric cancer.

Additionally, we validated two novel miRNAs which have been known as tumor suppressive (miR-655) and as oncogenic (miR-938) miRNA in gastrointestinal cancers other than gastric^37–39,49^. Although they did not show any stepwise elevation during multistep carcinogenesis, we could prove that they worked as tumor suppressive and oncogenic in gastric cancers. These results also prove that those novel miRNAs are important in gastric cancers, but do not work in multistep carcinogenesis, so they were not detected in our DEM miRNA groups.

Our study has several limitations. First, the sample size in miRNA array experiments is quite small (n = 24). To overcome this limitation, we replicated our microarray results in 77 FFPE samples and TCGA datasets. Secondly, the effects of altered miRNA on their target mRNA were not investigated. It is widely accepted that miRNAs have multiple -sometimes hundreds- of targets and the main approach to explore connections between miRNAs and their targets has been focused on the most significant target for each miRNA^50^. In the present study, we selected target genes that have been known to interact with miRNA in the previous studies by experimental investigation to confirm the relationship between miRNA and target mRNA. Further *in vitro* experiments using cell lines and larger scale studies are recommended to confirm our results and to analyze the potential effects of those deregulated miRNA.

In conclusion, we found that the upregulated (miR-107 and miR-300) or down-regulated (miR-26a and miR-375) miRNAs show alteration at early stages of gastric tumorigenesis and may be used as candidate biomarkers. Further investigation is needed to elucidate the exact role of these miRNAs in early gastric carcinogenesis.

## Materials and Methods

### Ethics statement

Fresh tumor and non-tumor samples were obtained by forceps biopsy at Samsung Comprehensive Cancer Center. Informed consent was obtained from all individuals who participated in this study. The study protocol was approved by the institutional review board of Samsung Medical Center (IRB 2010-09-020-008) and all experiments were performed in accordance with the approved guidelines and regulations.

### Samples

Fresh tumor samples from 14 patients were obtained during endoscopy by forceps biopsy and were used as a test set (seven well to moderately differentiated intestinal-type EGCs, three HGDs, and four LGDs) (Table 1). No patient had prior chemo or radiation therapy. All lesions were completely removed further with an endoscopic submucosal dissection technique after tissue acquisition with forceps biopsy, and the resected specimens were reviewed by two pathologists. The pathologic diagnosis of histological grade (LGD, HGD, or EGC) was made based on the review of both forceps biopsy and endoscopic submucosal dissection specimens to ensure histological homogeneity as previously described^3^. Tumors mixed with components of different histological grades were excluded from the study. Non-tumor gastric samples more than 2 cm apart from the tumor were used as a reference. All non-tumor samples showed intestinal metaplasia. All fresh tissue samples were snap frozen in liquid nitrogen immediately after collection and stored at −80 °C until use. *Helicobacter pylori* infection status was determined using both urea breath test and histology, while Epstein-Barr virus status was determined by the Epstein-Barr encoding region *in situ* hybridization.

In addition, a total of 77 formalin-fixed paraffin-embedded (FFPE) samples were collected and used as a validation set (21 well/moderately differentiated intestinal-type EGCs, 22 HGDs, 24 LGDs, and 10 non-tumor tissues). Informed consent was obtained from all individuals who participated in the study, and the study protocol was approved by the institutional review board.

### miRNA microarray by NanoString

For the nCounter analysis, 10 consecutive tissue sections (4-μm thick) from archival FFPE tissues of precursor lesions (LGD and HGD) and carcinoma were used. Total miRNA was isolated using the Qiagen miRNeasy Kit (Qiagen, Valencia, CA) according to the manufacturer’s protocol. Total miRNA samples were analyzed using the nCounter Human miRNA Expression Assay kit (NanoString, Seattle, WA) according to manufacturer’s instructions. Briefly, 100 ng RNA was incubated in the presence of miRNA-specific capture and reporter probes and non-hybridized probes were removed, followed by immobilization of the purified hybridized complexes. The abundance of specific target molecules was subsequently quantified on the nCounter Digital Analyzer by counting the individual fluorescent barcodes and assessing the target molecules, as previously described^51^.

### Differentially expressed miRNA analysis and correlation with mRNA

To identify the differentially expressed miRNAs (DEMs) among LGDs, HGDs, and EGCs, we calculated analysis of variance (ANOVA) p-value, average of miRNA expression within the same group, and fold change by comparing expression levels among the three groups. DEMs were defined by a p-value < 0.01, average expression >5 in at least one group, and |log2(fold change)| >1. The adjusted p-value was calculated using Benjamini and Hochberg algorithm in R. We next performed a k-means clustering to categorize DEMs into groups using expression patterns. To quantify the mRNA expression level, we counted the number of aligned fragments for each gene using HTSeq-0.6.1 with parameters (−s no, −r pos, −f bam, −m intersection-non-empty, and −t exon) according to the Ensembl transcript annotation (GRCh37 version) and calculated the fragments per kilobase per million mapped read (FPKM) values of each gene. Correlation between miRNA and mRNA expression was calculated by Spearman’s correlation using R. miRNA and mRNA expression data from The Cancer Genome Atlas (TCGA) datasets^52^ were also used to validate the patterns of DEMs and correlation between expression of miRNA and target mRNA expression.

### Real-time quantitative PCR analysis with FFPE samples

Ten serial paraffin cuts obtained in an Eppendorf tube were deparaffinized in xylene. Total RNA was isolated using RNeasy Micro Kit (Qiagen, Germany) according to the manufacturer’s instructions. RNA concentrations were measured using NanoDrop (Thermo Scientific, USA). Total RNA from each sample was reverse transcribed with the TaqMan MicroRNA Reverse Transcription kit (Thermo Fisher, USA). Reverse transcription was performed with the following thermal cycling parameters: 30 minutes at 16 °C, 30 minutes at 42 °C, and 5 minutes at 85 °C (Bio-Rad).

The miRNA expression was determined with TaqMan MicroRNA primer/probe sets. All qPCR reactions were performed with 7900 Fast Real-Time PCR System (Applied Biosystems, Foster City, CA). Gene expressions for hsa-miR-375 (Assay ID, 000564), hsa-miR-370 (Assay ID, 002275), hsa-miR-26a (Assay ID, 000405), hsa-miR-300 (Assay ID, 241035), hsa-miR-1260 (Assay ID, 002896), and hsa-miR-107 (Assay ID, 000443), hsa-miR-655 (Assay ID, 001612), and hsa-miR-938 (Assay ID, 002181) were quantified by TaqMan microRNA Assays (Applied Biosystems) according to manufacturers’ protocol and normalized by U6 snRNA (Assay ID 001973). PCR amplification of target genes and quantification of PCR products were performed by ABI PRISM 7900 HT Sequence Detection System (Applied Biosystems). Differences in the expression were determined by the relative quantification method; the Ct values of the test genes were normalized to the Ct values of the endogenous control U6 snRNA. The fold change was calculated using the equation 2−ΔΔCt.

## Electronic supplementary material

Supplementary information
