# Supplementary material for: MicroRNA Expression Profiles in Gastric Carcinogenesis
Source: Sci Rep. 2018 Sep 26;8:14393. doi: 10.1038/s41598-018-32782-8 (PMC6158169; doi:10.1038/s41598-018-32782-8)
Supplement: Supplementary file 1 — Supplementary information [file 41598_2018_32782_MOESM1_ESM.docx]

MicroRNA Expression Profiles in Gastric Carcinogenesis

Jinha Hwang^1,+^, Byung-Hoon Min^2,+^, Jiryeon Jang^3,4,+^, So Young Kang^4^, Hyunsik Bae^4^, Se Song Jang^1^, Jong-Il Kim^1,5,6,*^, Kyoung-Mee Kim^4,*^


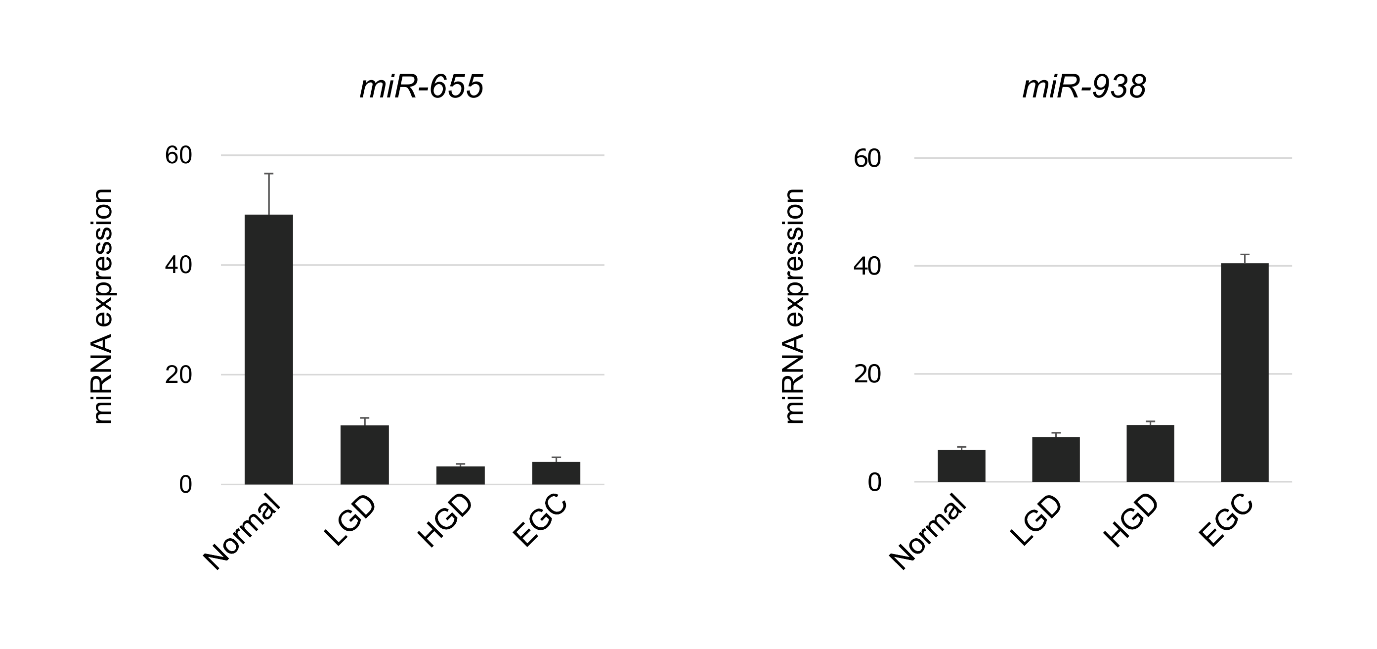
Supplementary Figure1. The expression levels of two novel miRNAs (miR-655 and miR-938) in normal gastric mucosa, adenoma and carcinoma measured by RT-qPCR.


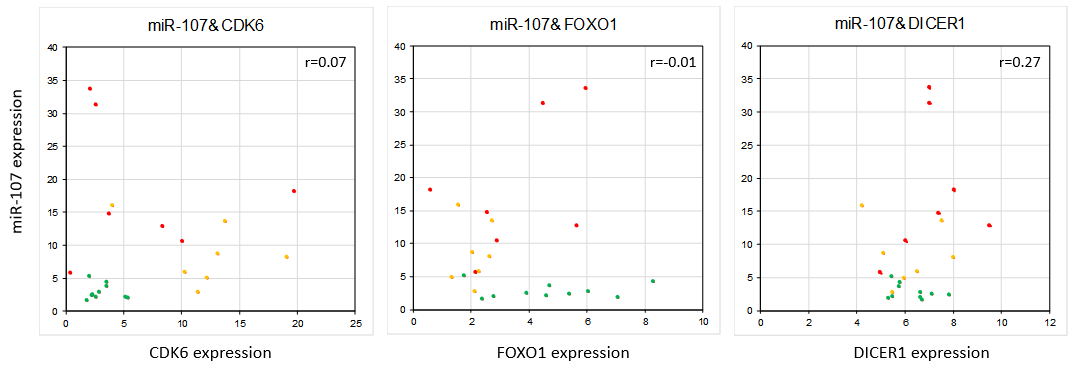


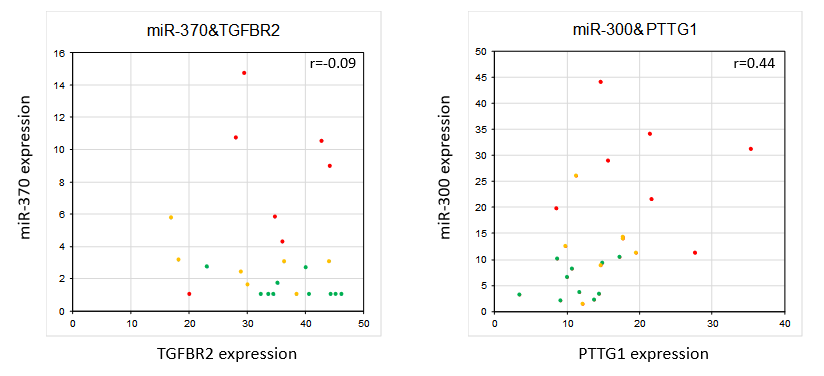


Supplementary Figure2. Spearman’s correlation between miRNAs (mir-107, mir370, and mir-300) and well-known target genes in samples from normal, LGD, HGD, and EGC.

Supplementary Table 1. The list of differentially expressed miRNA (fold change >= 2.5, p-value < 0.01)

| **DEM group** | **miRNA** | **adenoma/normal** | **egc/normal** | **egc/adenoma** | **pvalue** | **FDR** |
| --- | --- | --- | --- | --- | --- | --- |
| DEM-1a | hsa-miR-1274a | 0.14 | 0.05 | 0.38 | 1.43E-07 | 5.25E-05 |
|  | hsa-miR-150 | 0.21 | 0.08 | 0.38 | 2.54E-03 | 1.86E-02 |
|  | hsa-miR-1979 | 0.23 | 0.19 | 0.86 | 2.06E-04 | 4.57E-03 |
|  | hsa-miR-26a | 0.33 | 0.07 | 0.22 | 1.39E-07 | 5.25E-05 |
|  | hsa-miR-375 | 0.45 | 0.15 | 0.33 | 6.32E-04 | 9.73E-03 |
|  | hsa-miR-125a-5p | 0.54 | 0.37 | 0.68 | 1.76E-03 | 1.54E-02 |
|  | hsa-miR-1260 | 0.34 | 0.15 | 0.45 | 2.01E-05 | 1.84E-03 |
|  | hsa-miR-1274b | 0.21 | 0.13 | 0.62 | 5.51E-07 | 1.26E-04 |
|  | hsa-miR-145 | 0.24 | 0.20 | 0.85 | 4.14E-05 | 1.98E-03 |
|  | hsa-miR-193b | 0.15 | 0.20 | 1.34 | 8.66E-04 | 1.14E-02 |
|  | hsa-miR-1975 | 0.24 | 0.20 | 0.85 | 3.24E-05 | 1.98E-03 |
|  | hsa-miR-423-3p | 0.37 | 0.35 | 0.95 | 2.12E-04 | 4.57E-03 |
|  | hsa-miR-484 | 0.24 | 0.65 | 2.66 | 5.16E-03 | 2.46E-02 |
|  | hsa-miR-532-3p | 0.24 | 0.32 | 1.35 | 1.61E-04 | 4.38E-03 |
|  | hsa-miR-574-3p | 0.32 | 0.26 | 0.80 | 2.11E-06 | 2.58E-04 |
|  | hsa-miR-720 | 0.49 | 0.37 | 0.76 | 6.85E-07 | 1.26E-04 |
| DEM-1b | hsa-let-7a | 0.80 | 0.36 | 0.45 | 1.14E-03 | 1.30E-02 |
|  | hsa-let-7g | 1.47 | 0.58 | 0.39 | 9.75E-03 | 3.03E-02 |
|  | hsa-miR-16 | 0.73 | 0.22 | 0.31 | 5.87E-03 | 2.66E-02 |
|  | hsa-miR-200b | 1.56 | 0.57 | 0.37 | 8.32E-03 | 2.87E-02 |
|  | hsa-miR-200c | 0.73 | 0.26 | 0.36 | 1.87E-04 | 4.43E-03 |
|  | hsa-miR-21 | 1.56 | 0.55 | 0.35 | 1.79E-04 | 4.38E-03 |
|  | hsa-miR-29a | 0.91 | 0.39 | 0.43 | 2.22E-04 | 4.60E-03 |
| DEM-2 | hsa-miR-126 | 2.86 | 1.65 | 0.58 | 2.79E-03 | 1.86E-02 |
|  | hsa-miR-194 | 2.99 | 0.61 | 0.20 | 5.53E-05 | 2.26E-03 |
|  | hsa-miR-200a | 5.47 | 2.31 | 0.42 | 8.36E-06 | 8.77E-04 |
| DEM-3 | hsa-miR-106a+hsa-miR-17 | 4.50 | 7.47 | 1.66 | 8.79E-03 | 2.96E-02 |
|  | hsa-miR-107 | 2.98 | 6.31 | 2.12 | 2.73E-04 | 5.14E-03 |
|  | hsa-miR-10a | 1.71 | 2.71 | 1.58 | 7.83E-03 | 2.77E-02 |
|  | hsa-miR-10b | 1.92 | 4.90 | 2.55 | 1.24E-03 | 1.33E-02 |
|  | hsa-miR-1253 | 2.90 | 4.37 | 1.51 | 4.56E-03 | 2.27E-02 |
|  | hsa-miR-1254 | 2.13 | 5.42 | 2.55 | 9.25E-03 | 2.96E-02 |
|  | hsa-miR-140-5p | 1.58 | 3.56 | 2.26 | 7.41E-03 | 2.76E-02 |
|  | hsa-miR-142-5p | 2.18 | 3.41 | 1.56 | 4.29E-03 | 2.23E-02 |
|  | hsa-miR-181b+hsa-miR-181d | 2.18 | 3.84 | 1.76 | 3.29E-03 | 1.99E-02 |
|  | hsa-miR-18a | 2.03 | 3.46 | 1.70 | 3.29E-04 | 5.89E-03 |
|  | hsa-miR-1913 | 2.10 | 5.47 | 2.61 | 2.95E-03 | 1.91E-02 |
|  | hsa-miR-192 | 7.98 | 10.95 | 1.37 | 5.96E-03 | 2.66E-02 |
|  | hsa-miR-302a | 2.01 | 5.44 | 2.71 | 6.70E-03 | 2.66E-02 |
|  | hsa-miR-329 | 3.96 | 4.93 | 1.25 | 4.11E-03 | 2.22E-02 |
|  | hsa-miR-340 | 1.07 | 3.36 | 3.15 | 7.18E-03 | 2.70E-02 |
|  | hsa-miR-373 | 1.47 | 3.78 | 2.58 | 3.89E-03 | 2.13E-02 |
|  | hsa-miR-410 | 1.07 | 3.76 | 3.52 | 9.15E-05 | 3.23E-03 |
|  | hsa-miR-517c+hsa-miR-519a | 2.04 | 4.13 | 2.02 | 1.42E-03 | 1.39E-02 |
|  | hsa-miR-518d-3p | 1.72 | 3.78 | 2.20 | 1.04E-04 | 3.23E-03 |
|  | hsa-miR-518e | 3.63 | 5.40 | 1.49 | 1.30E-03 | 1.37E-02 |
|  | hsa-miR-542-3p | 2.05 | 2.75 | 1.34 | 3.87E-03 | 2.13E-02 |
|  | hsa-miR-548a-3p | 1.76 | 2.97 | 1.69 | 8.61E-04 | 1.14E-02 |
|  | hsa-miR-548b-3p | 1.60 | 3.65 | 2.28 | 5.49E-03 | 2.55E-02 |
|  | hsa-miR-548d-5p | 2.54 | 4.04 | 1.59 | 1.60E-03 | 1.47E-02 |
|  | hsa-miR-548e | 2.21 | 5.22 | 2.36 | 9.41E-03 | 2.99E-02 |
|  | hsa-miR-548f | 1.74 | 4.04 | 2.32 | 3.59E-03 | 2.09E-02 |
|  | hsa-miR-548g | 2.09 | 3.08 | 1.47 | 1.74E-03 | 1.53E-02 |
|  | hsa-miR-555 | 2.63 | 5.18 | 1.97 | 5.70E-03 | 2.61E-02 |
|  | hsa-miR-572 | 1.93 | 4.79 | 2.49 | 2.46E-03 | 1.83E-02 |
|  | hsa-miR-601 | 2.53 | 4.69 | 1.85 | 3.96E-05 | 1.98E-03 |
|  | hsa-miR-632 | 1.32 | 5.51 | 4.17 | 6.36E-04 | 9.73E-03 |
|  | hsa-miR-646 | 1.21 | 3.62 | 2.98 | 2.69E-04 | 5.14E-03 |
|  | hsa-miR-651 | 1.98 | 4.04 | 2.04 | 1.44E-04 | 4.23E-03 |
|  | hsa-miR-658 | 2.14 | 5.06 | 2.37 | 7.91E-03 | 2.78E-02 |
|  | hsa-miR-671-3p | 1.33 | 4.22 | 3.17 | 1.59E-03 | 1.47E-02 |
|  | hsa-miR-759 | 2.23 | 4.06 | 1.82 | 9.19E-03 | 2.96E-02 |
|  | hsa-miR-760 | 3.56 | 6.36 | 1.79 | 9.67E-03 | 3.03E-02 |
|  | hsa-miR-877 | 1.97 | 4.67 | 2.37 | 4.21E-03 | 2.22E-02 |
|  | hsa-miR-892b | 1.91 | 3.31 | 1.73 | 8.91E-03 | 2.96E-02 |
|  | hsa-miR-933 | 1.62 | 5.11 | 3.16 | 9.12E-03 | 2.96E-02 |
|  | hsa-miR-98 | 2.39 | 4.04 | 1.69 | 2.41E-03 | 1.82E-02 |
|  | ebv-miR-BART1-3p | 1.65 | 2.83 | 1.71 | 1.68E-04 | 4.38E-03 |
|  | ebv-miR-BART16 | 2.76 | 5.99 | 2.17 | 1.51E-03 | 1.44E-02 |
|  | ebv-miR-BART6-5p | 2.09 | 3.07 | 1.47 | 9.77E-04 | 1.18E-02 |
|  | kshv-miR-K12-4-5p | 2.15 | 3.19 | 1.48 | 2.33E-03 | 1.80E-02 |
|  | hsa-miR-1182 | 2.16 | 6.51 | 3.01 | 1.90E-03 | 1.58E-02 |
|  | hsa-miR-1231 | 2.25 | 5.79 | 2.57 | 5.22E-05 | 2.25E-03 |
|  | hsa-miR-1255a | 3.85 | 8.70 | 2.26 | 1.64E-03 | 1.49E-02 |
|  | hsa-miR-1287 | 2.03 | 4.46 | 2.20 | 6.66E-03 | 2.66E-02 |
|  | hsa-miR-137 | 2.30 | 5.02 | 2.19 | 1.09E-03 | 1.29E-02 |
|  | hsa-miR-1470 | 1.92 | 8.69 | 4.54 | 1.22E-03 | 1.33E-02 |
|  | hsa-miR-1537 | 3.14 | 5.74 | 1.83 | 2.88E-03 | 1.90E-02 |
|  | hsa-miR-1825 | 2.16 | 4.35 | 2.01 | 8.95E-03 | 2.96E-02 |
|  | hsa-miR-193a-3p | 2.16 | 4.98 | 2.31 | 4.32E-05 | 1.98E-03 |
|  | hsa-miR-1973 | 2.27 | 7.38 | 3.26 | 2.79E-03 | 1.86E-02 |
|  | hsa-miR-34c-3p | 1.56 | 4.57 | 2.94 | 2.89E-04 | 5.30E-03 |
|  | hsa-miR-370 | 2.02 | 5.65 | 2.80 | 1.77E-04 | 4.38E-03 |
|  | hsa-miR-452 | 2.31 | 5.25 | 2.27 | 1.26E-03 | 1.34E-02 |
|  | hsa-miR-453 | 1.71 | 7.03 | 4.12 | 4.88E-03 | 2.36E-02 |
|  | hsa-miR-486-3p | 2.98 | 6.90 | 2.31 | 8.65E-05 | 3.23E-03 |
|  | hsa-miR-490-5p | 2.44 | 8.99 | 3.68 | 8.10E-04 | 1.12E-02 |
|  | hsa-miR-508-5p | 1.68 | 6.45 | 3.85 | 8.71E-04 | 1.14E-02 |
|  | hsa-miR-519e | 1.31 | 6.27 | 4.79 | 3.09E-03 | 1.94E-02 |
|  | hsa-miR-548a-5p | 2.36 | 4.42 | 1.87 | 8.09E-03 | 2.81E-02 |
|  | hsa-miR-551a | 2.57 | 8.55 | 3.33 | 2.66E-03 | 1.86E-02 |
|  | hsa-miR-554 | 1.84 | 5.23 | 2.85 | 9.66E-03 | 3.03E-02 |
|  | hsa-miR-591 | 2.59 | 6.03 | 2.32 | 4.69E-03 | 2.28E-02 |
|  | hsa-miR-620 | 2.64 | 8.06 | 3.05 | 5.52E-04 | 9.00E-03 |
|  | hsa-miR-623 | 2.28 | 4.43 | 1.94 | 1.40E-03 | 1.39E-02 |
|  | hsa-miR-655 | 2.90 | 7.12 | 2.45 | 2.91E-05 | 1.98E-03 |
|  | hsa-miR-767-3p | 1.87 | 6.40 | 3.43 | 3.53E-03 | 2.07E-02 |
|  | hsa-miR-874 | 3.40 | 8.71 | 2.56 | 9.40E-04 | 1.17E-02 |
|  | hsa-miR-886-5p | 1.57 | 7.87 | 5.00 | 7.06E-03 | 2.67E-02 |
|  | hsa-miR-889 | 3.28 | 4.71 | 1.44 | 5.32E-03 | 2.51E-02 |
|  | hsa-miR-938 | 1.87 | 7.08 | 3.78 | 1.05E-04 | 3.23E-03 |
|  | hsa-miR-1297 | 1.11 | 3.34 | 3.02 | 2.33E-03 | 1.80E-02 |
|  | hsa-miR-1308 | 1.22 | 3.55 | 2.92 | 1.97E-04 | 4.51E-03 |
|  | hsa-miR-1915 | 1.22 | 3.89 | 3.20 | 4.23E-03 | 2.22E-02 |
|  | hsa-miR-203 | 1.99 | 3.50 | 1.76 | 8.74E-03 | 2.96E-02 |
|  | hsa-miR-206 | 1.90 | 2.84 | 1.50 | 2.98E-03 | 1.91E-02 |
|  | hsa-miR-224 | 2.15 | 5.34 | 2.48 | 2.38E-03 | 1.82E-02 |
|  | hsa-miR-300 | 2.13 | 4.58 | 2.15 | 3.17E-05 | 1.98E-03 |
|  | hsa-miR-346 | 1.50 | 3.08 | 2.05 | 4.20E-03 | 2.22E-02 |
|  | hsa-miR-431 | 1.61 | 2.90 | 1.80 | 9.59E-03 | 3.03E-02 |
|  | hsa-miR-519b-3p | 1.66 | 3.21 | 1.93 | 1.88E-03 | 1.58E-02 |
|  | hsa-miR-542-5p | 1.89 | 5.55 | 2.93 | 4.67E-03 | 2.28E-02 |
|  | hsa-miR-548d-3p | 1.09 | 4.39 | 4.02 | 8.74E-03 | 2.96E-02 |
|  | hsa-miR-589 | 1.81 | 6.29 | 3.48 | 1.55E-06 | 2.28E-04 |
|  | hsa-miR-96 | 1.92 | 2.62 | 1.36 | 1.04E-04 | 3.23E-03 |
|  | ebv-miR-BART9 | 1.55 | 3.51 | 2.26 | 2.25E-04 | 4.60E-03 |
|  | hcmv-miR-UL22A | 1.89 | 3.95 | 2.09 | 7.81E-03 | 2.77E-02 |
|  | hsa-miR-100 | 0.84 | 2.11 | 2.51 | 6.94E-03 | 2.66E-02 |
|  | hsa-miR-1268 | 1.33 | 6.00 | 4.53 | 6.50E-03 | 2.66E-02 |
|  | hsa-miR-323-3p | 1.40 | 5.60 | 4.01 | 4.23E-03 | 2.22E-02 |
|  | hsa-miR-324-5p | 0.58 | 2.56 | 4.38 | 3.32E-03 | 1.99E-02 |
|  | hsa-miR-548j | 1.60 | 3.21 | 2.00 | 7.95E-03 | 2.78E-02 |
|  | hsa-miR-654-5p | 1.37 | 5.62 | 4.11 | 1.03E-04 | 3.23E-03 |
|  | ebv-miR-BHRF1-1 | 2.09 | 2.62 | 1.26 | 4.95E-03 | 2.37E-02 |
